# The Impact of Religion and Provision of Information on Increasing Knowledge and Changing Attitudes to Organ Donation: An Intervention Study

**DOI:** 10.1007/s10943-019-00961-0

**Published:** 2019-12-14

**Authors:** Ferid Krupic

**Affiliations:** 1 Department of Orthopaedics, Institute of Clinical Sciences, The Sahlgrenska Academy, University of Gothenburg, Göteborgsvägen 31, 431 80 Mölndal, Sweden; 2 Department of Anaesthesiology and Intensive Care, Institute of Clinical Sciences, The Sahlgrenska Academy, University of Gothenburg, Göthenburg, Sweden

**Keywords:** Organ donation, Information, Religion, Class, Education, Intervention, Qualitative research

## Abstract

One of the most significant developments in recent history has probably been organ donation and organ transplantation. They are frequently the only treatment available in certain cases. However, there is an ever-increasing discrepancy between the number of people needing transplantation and the organs available, because the decision to donate an organ is up to each individual. The study aims to assess the impact of the intervention on knowledge, attitudes and practices on organ donation among religious immigrants in Sweden. Data were collected through three group interviews using open-ended questions and qualitative content analysis. Thirty-six participants, 18 males and 18 females from six countries, participated in the focus group interviews. The analysis of the collected data resulted in two main categories: “Religion in theory and practice” and “More information—more knowledge about organ donation” including seven subcategories. Understanding of religion and religiosity, happiness by taking the class, the practice of religion in everyday life, the overcoming the prejudices in religion, having more information about organ donation and the donations process, as well as that the increased information changes people’s minds, were some of things the informants emphasised as predictors of the decision of organ donation. A class dealing with religion, the religious aspects of organ donation and the way the Swedish healthcare system is organised increased people’s knowledge and changed their attitudes so they became potential organ donors. More intervention studies are needed in every field of medicine to build confidence and give time to educate and discuss issues with potential organ donors in Sweden.

## Introduction

One of the most significant developments in recent history has probably been organ donation (OD) and organ transplantation (OT). They are frequently the only treatment available in certain cases. However, even though, in terms of feasibility, transplantation is increasingly accessible, there is an ever-increasing discrepancy between the number of people needing transplantation and the organs available (Levitt 2015). Healthcare professionals play an important role in the decision to donate organs (Council of Europe 2012). However, the decision to donate an organ is up to each individual. For the younger generation, the whole concept is still very vague and often misunderstood. Unfortunately, the figures show that we could do a great deal more to help those whose lives depend on transplantation (Wilow 2013). In Sweden, the organs of 17.6 per million deceased people are donated in 2018 (The Swedish Health and Medical Services Act 2014). This is a much lower figure than in other countries. The number of deceased organ donors in Europe varies between countries. Spain and Croatia have the largest number of donors (pmp) in Europe, with 34.0 and 39.2 donors, respectively (Report of the Madrid Consultation 2011; Citerio et al. 2016). However, Bosnia and Herzegovina and Kosovo, at 2.0 and 1.2 donors, have the lowest (Citerio et al. 2016). It has been shown that a large number of issues affect a person’s decision to donate organs. Religion has been shown to have a significant influence on this decision in a number of studies (Randhawa 1998; Hayward and Madill 2003; Alkhawari et al. 2005; Davis and Randhawa 2006). Studies of the major religions among African-Caribbeans and South-Asians in the UK—Islam, Hinduism, Sikhism, Buddhism and Christianity—have been undertaken. These religions are not opposed to organ donation, but within them there are differences of opinion. However, people base their decisions in this matter on their religious convictions (Randhawa 1998; Oliver et al. 2011). The belief that saving life is most important is common to all religions, so all the mainstream religions support organ donation (Bruzzone 2008). Duzenly (2005) explains that Islam permits organ and tissue transplantation in order to save human life or vital organs. The impact of race and ethnicity (Siminoff et al. 2006; Morgan et al. 2006), gender (Chen et al. 2006) and age (Miles and Frauman 1998; Conesa et al. 2003) has been studied. However, it has been shown that, as a rule, higher levels of education and income (Conesa et al. 2003, 2006; Rumsey et al. 2003) lead to higher levels of deceased organ donation (Rumsey et al. 2003). Education and studies are therefore needed to increase the number of people willing to donate their organs. This study is based on the hypothesis that education on religions and their view of organ donation should increase participants’ understanding and improve their stance regarding organ donation.

## Aim of the Study

The present study aimed to assess the impact of the intervention on knowledge, attitudes and practices on organ donation among religious immigrants in Sweden.

## Methods

### Design

The study was designed as a qualitative study using data from interviews with participants from Bosnia and Herzegovina, Macedonia, Turkey, Lebanon, Slovenia and Kosovo. The data were collected through three focus group interviews (McLafferty 2004), with 12 participants, six women and six men, in each group.

### Participants

The inclusion criteria were participants from Bosnia and Herzegovina, Macedonia, Turkey, Lebanon, Slovenia and Kosovo, who were more than 20 years old, had lived in Sweden for more than 10 years and described themselves as religious. Forty-nine participants took a class on their religion and organ donation. The class lasted 4 h and was organised by the Bosnian and Somalian association in Gothenburg. One lecturer was the head imam for the western part of Sweden, who spoke about religion and its impact on organ donation and transplantation. The other lecturer was the author of the study who lectured on the process when a person decides to donate organs and how the Swedish healthcare system works in this case. The class took place on Friday, 30 November 2018. The three interviews took place in groups the following year, with about one interview per month. Thirty-six participants participated in the interviews, 18 women and 18 men, aged 29–71 years (mean 50.0 years). The men were aged 36–72 (mean 54.0 years) and the women 41–60 (mean 50.5 years). The participants were assigned to three age groups; the first, with twelve participants, aged 34–62 years (six women and six men), the second, with twelve participants, aged 40–65 years (six women and six men), and the third, with nine participants, aged 60–73 years (six women and six men). The interviews and all the communication were carried out in the Bosnian and Swedish languages. The demographic and clinical characteristics of the informants are shown in Table 1.

**Table 1:** Characteristics of the study population

Variables	Numbers
Gender	
Male	18
Female	18
Total	36
Educational level	
Elementary school	7
High school	19
University	10
Total	36
Age (years)	
≤ 30	3
31–40	8
41–50	7
51–60	13
61–70	3
≥ 70	2
Total	36
Countries of birth	
Bosnia and Herzegovina	17
Macedonia	4
Turkey	2
Lebanon	5
Slovenia	3
Kosovo	5
Total	36
Religion	
Islam	22
Christian orthodox	0
Other	0
Total	36

### Procedure

Data were collected through group interviews by the first author, using individualised open-ended questions, following an interview guide inspired by Kvale (1997). The interviews were performed from November 2018 to March 2019. They began with small talk. The opening questions were “What do you know about the religious impact on organ donation?”, “Would you consider donating your own organs or organs from a member of your family?” and “Have you changed your opinion on organ donation after attending the class about religion and organ donation?”. The initial questions were supplemented with other short questions, such as “Could you please tell me more about that?” and “What do you mean by that?”. All contact with the participants was organised in collaboration with a key person in a Bosnia and Herzegovina and Somalia association in the western part of Sweden. Participants who attended the class and met the inclusion criteria were asked to participate in the study on the same day after the class. When the key person had recruited enough participants, the author of the study was contacted and the interview was arranged. Printed information about the aim and background of the study was distributed to the participants and repeated to them orally before the interview. The interviews were carried out in groups of twelve participants and held in the facilities of the Bosnian and Somalian association. The interviews were carried out in Bosnian and Swedish by the author of the study, who is bilingual. Some younger participants chose to speak Swedish. All the interviews were therefore translated first into Swedish by the author, after which a professional translator checked the translation. The interviewer only interrupted to ask questions or to follow-up on the information given. All the participants gave their signed informed consent before the interviews. The interviews lasted between 55 and 105 min and were taped and transcribed verbatim.

### Data Analysis

The qualitative content analysis method, in accordance with Graneheim and Lundman (2004), was chosen for the analysis and interpretation of the collected data. This method is suitable for the analysis of qualitative data because, using this method, the researcher is able to condense a large amount of data into a small number of codes, subcategories, categories and themes. The author conducted a manifest analysis of the text. The transcripts were read carefully in order to identify the informants’ experiences and conceptions. The analysis then proceeded by extracting meaningful units, consisting of one or several words, sentences, or paragraphs, containing aspects related to each other and addressing a specific topic in the material. Meaningful units, related to each other through their content and context, were then abstracted and grouped together into a condensed meaningful unit, with a description close to the original text. The condensed text was further abstracted and labelled with a code. Codes that addressed similar issues were then grouped together, resulting in subcategories. Subcategories that focused on the same problem were brought together, in order to create more extensive conceptions, which addressed an obvious issue (Graneheim and Lundman 2004). The results are presented with direct quotations from the interviews.

### Ethics

Since there was no physical intervention and no information on individual health issues was involved in the study, there was no need to involve the ethical board, according to Swedish law (2015). The World Medical Association Declaration of Helsinki (1964) was followed carefully. The informants’ identities were protected, i.e. their names and personal identity numbers were not stated in the recordings or any publications. The audiotapes used for the interviews were stored in a locked safe at the hospital. The identity of the participants could therefore not be traced. The study information given to the participants included its voluntary nature and the fact that they could withdraw at any time without incurring penalties or losing access to services.

## Results

The analysis of the text resulted in two main categories and seven subcategories, based on the participants’ description of their situation regarding OD. The categories, together with the subcategories, are presented in Table 2. The categories were religion in theory and practice and how more access to information increases knowledge of OD.

**Table 2:** Overview of the theme, categories and subcategories

Categories	Subcategories	Theme
Religion in theory and practice	Religion as a concept	Education about religion and provision of information as predictors of organ donation
	Religion in everyday practice	
	Prejudice in religion	
	Information about the Swedish healthcare system	
More information—more knowledge about organ donation	More information leads to use of donor cards	
	I own my body	
	More information changes people’s minds about organ donation	

### Religion in Theory and Practice

All the participants in the study described themselves as religious and practising religion on a daily basis. They also pointed out that their thinking, their world view, regarding the religious aspect of organ donation, changed fundamentally after the class. They described their thoughts about this as cheerful, more complete and more fundamental and said they were more confident about these matters than a few months before. They also emphasised that the class had increased their understanding of religion in general, as well as what their religion said, and that they thought about organ donation within the family, with other people, or the donation of parts of organs. Regarding theoretical and practical issues in religion, all the respondents explained that these matters were clearer after the class. All of them also stressed that, since the class, they could all be referred to as religious and future donors of their organs, in the right sense of the word.

### Religion as a Concept

All the subjects in this study imagined the theory of religion differently before the class. Their understanding of religion was most often based on earlier experiences and not on a scientific basis or information from learned people educated in the faith. Their sense of faith, depth of faith, notion of religion and religiosity were also based on earlier experiences acquired from their parents or grandparents. All the respondents in the study stated that their understanding of religion and religiosity had changed fundamentally and that their understanding of religion was quite different after the class. Everyone expressed happiness that they had taken the class, as well as the need for reunion and classes in other subjects.

“When I compare myself then and today… I am a different person in terms of religion and religiosity”.

“I thought I knew a lot about our religion, but we all learn while we are alive. Thank you”.

### Religion in Everyday Practice

All the respondents in the study emphasised that the breadth of their comprehension of religion also changed in terms of the practice of religion in everyday life. They pointed out that their practice of religion and their relationship to faith had changed in practical terms. They explained that their belief and the meaning of faith were now based on donating their organs to others, being more humane and helping their brothers in difficulties, as well as helping mankind deal with medical problems. Their perception of religion before the class in a practical sense was based on saying their daily prayers, being good people and not doing anything that would harm others. However, all the respondents concluded that this was not actually enough and that the class had opened their eyes. By acquiring knowledge and information from the class, they realised that they were happier in their faith and that faith is not just prayer and knowledge or knowing religious language. Faith and being a true believer according to the respondents also involves taking care of others, helping at a given moment and donating your organs to others.

“I never thought about donating organs and religion, I didn’t know much about it, but I know now and now I would definitely donate my organs to others”.

“I am actively thinking about organ donation after the class… I never thought about it before, but today I would absolutely donate my body to others”.

### Prejudice in Religion

Prejudice has always been an important aspect of religion, and it sometimes has to be overcome to explain some aspects of religion and the religious view of organ donation. After completing the class, all the respondents in the study were confident in themselves and the opinion that Islam as a religion allows the donation of organs to their families, as well as to other non-family members, and even to persons who are not of the same faith as the donor. All of them were also convinced that the prejudices regarding religion and organ donation must be overcome and that they have no basis in the modern era of medical and religious progress. All the respondents’ awareness of prejudice and organ donation also increased.

“I have heard various things about our faith and organ donation, but I know now that no one else can tell me what is right and what is not”.

“I had heard that, when a kidney is donated, it isn’t possible to urinate after death. That’s ridiculous. I’m now convinced that it’s nonsense”.

### More Information: More Knowledge About Organ Donation

All the respondents in the study of the issue of religion and organ donation and information about the way the Swedish healthcare system functions had only words of praise. Apart from the religious part of their lives, most of the respondents were employed; most of them “rushed” through life and had not even considered the things they heard in the class before in their lives. All the respondents also emphasised that they are confident in their thoughts, they are “in their own territory”, they know what they are doing and they are fully aware of how and what they are doing about religion and the Swedish healthcare system. All those surveyed in the study were, however, disappointed that so little was known about things that are so important to people’s lives and that it is very difficult to obtain true, reliable information. All the respondents expressed their deep appreciation that they had had the chance to take the class.

### Information About the Swedish Healthcare System

The majority of respondents learned about the way the medical system in Sweden and organ donation function for the first time by attending the class. Even though they had spent at least 10 years living in Sweden, they learned for the first time in the class what to do when deciding to donate organs, the process and time of organ donation, as well as how organ donation works and the time of recovery after organ donation. Most respondents had not paid any attention to this important matter, although some of them had some idea but were not sure how to register for organ donation. All the respondents were happy that they now know how they can donate their organs and the way the Swedish healthcare system functions.

“I’m not so knowledgeable and I don’t know which rights I have in this country, but now I have information about everything and I’m very happy about it”.

“I didn’t do a lot of things and I didn’t ask for a doctor’s help, because I didn’t know how healthcare works in the medical field. Now I know and I’m glad to do it”.

### More Information Leads to Use of Donor Cards

The issue of a donor card for all the subjects was more of a problem than a benefit. Ignorance and lack of information about donor cards was clearly expressed by all the subjects in this study. Most of them emphasised that they had found the idea of a donor card very frightening before they took the class, because they had the wrong conception of what it meant and did not have full information about what a donor card is and what its benefits are. However, having learned about all the benefits of donor cards, all the respondents emphasised that they are holders of donor cards.

“I thought that, if I had a donor card, when I die, all my organs can be taken, but that’s not the case”.

“I now know that, even though I have a donor card, I determine which organ I want and do not want to donate”.

### I Own My Body

The class taken by the respondents in this study also pointed out and introduced the fact that everyone owns their own body and has the right to do precisely what they want with their body and organs. This fact, that everyone has a right to do what they want with their body and that every person owns their body, was welcomed as positive by all the respondents. They were happy to take the class and obtain new and important knowledge.

“Earlier, when I went to the doctor, I listened to him carefully and did everything he told me. Today, when I know my rights and duties, I think that I will consider everything the doctor tells me carefully”.

“When I think about all my visits to the doctor, if I had known what I know today, I would have done things differently”.

### More Information Changes People’s Minds About Organ Donation

All the respondents in this study expressed great satisfaction about completing the class, that they had obtained knowledge about organ donation and the way the Swedish healthcare system functions, that they had a donor card and had the chance to save other people’s lives. All the respondents said that they felt complete in their knowledge and important in society and the state, because they could help and knew what to do about donating organs to others. They had learned what and how to do it, if things went as they had planned or if there were any complications. They all stressed repeatedly that this class had turned their life and thinking around and they were grateful to God and the teachers who gave such a good, detailed class. They also stressed the importance of these classes and the need to repeat them more often.

“With this class, everything has fallen into place. I now have a donor card, I know what to do when I donate organs and I know how the healthcare system works”.,

“I’m happy to have met you, I’m happy to know how I can help others, I’m happy because I will be able to donate my body, thank you”.

“Man, where were you until now?”

## Discussion

The present study is the first intervention study in Sweden to investigate the relationship between the provision of more information about religion and the Swedish healthcare system and a direct increase in knowledge and a change in attitudes to organ donation. This is also the first study to include informants from six different countries and two different continents. The results of the study reveal several of the informants’ characteristics. There was one group of 36 participants, half of them were women with a mean age of 50 years and the majority of them had completed high-school education. All of them spoke Swedish and English, and the participants from the Balkans spoke Bosnian. Organ donation is affected by the availability of information, education about donation and religious factors, fear, prejudice and beliefs. After a class lasting 4 h, covering the topic of the effect of religion on attitudes to organ donation and the way the Swedish healthcare system functions, all the respondents changed their minds about organ donation and expressed their willingness to be a donor. They all said the information they had received was valuable and had helped them to see things differently. Unfortunately, to the author’s knowledge, there are no other similar studies in the world regarding the impact of information and education relating to religion and organ donation. In a previous study, this author found that, although the subjects were generally well educated, they held on to their donation priorities, where family members came first, followed by their friends and finally anyone else (Krupic et al. 2018). This study has surprising results because previous studies have shown that religious people often have a negative attitude to organ donation (Barcellos et al. 2005; Irving et al. 2012). The subjects in this study pointed out that their bodies were their own and they had a right to do whatever they thought best with them. However, after examining the subject in this study, they all stated that they would be willing to donate their organs, with no list of priorities, to anyone who needed them. In another study comprising 499 teachers from Bosnia and Herzegovina, of the three major religions (Islam, Roman Catholicism and Serbian Orthodox), the majority stated that they were positive about organ donation, from both living and deceased donors. There was a significant difference between the religious groups in relation to this issue (*p* = 0.0063). The majority also stated that they would donate an organ from a deceased member of their family, but a large number also said they were uncertain. Here, there was no significant difference between the religious groups (*p* = 0.7694). When asked to whom they would donate an organ, the majority replied that they would donate to a close relative but least of all to someone they did not know. There was a significant difference in the answers to this question between the groups (*p* = 0.0002) (Sadic et al. 2016). The informants said that they had obtained their knowledge about this from their parents, without any religious instruction on the issue. This may be the reason for the confusion and problems that people who have not been educated in their religion experience and why they refuse organ donation. It is necessary for people to learn what their religion says about organ donation. It was expected that they would be positive to OD, on the basis of other studies examining the issues associated with a positive opinion of OD, such as education, social standing and age (Ashraf et al. 2005; Mossialos et al. 2008; Krupic et al. 2017; Sadic et al. 2016). These studies also showed that it is necessary to instruct people about organ donation so they are willing to donate to anyone, not just to family members. A positive stance regarding OD was found to be related to their religious score (*p* = 0.015), marital status (*p* = 0.031) and knowledge score (*p* = 0.003). Employment and an understanding that religion permits OD were related to having greater knowledge, while a positive stance was linked to being single and having greater knowledge (Abukhaizaran et al. 2018). The class helped all the subjects to be more confident in themselves and also in their faith (Islam), which permits organ donation within families, but also to others, even those of a different faith. All the subjects in this study also believed that there is no foundation for any prejudice on the basis of religion regarding OD in the modern age. The first thing the subjects mentioned in the discussion was their previous lack of knowledge about OD and the Swedish healthcare system and they stressed how important that knowledge is, because a lack of knowledge meant it was unlikely they would participate in OD. This supports the findings of other studies, worldwide, where the level of knowledge was shown to predict people’s stance regarding OD. Those with more knowledge were more likely to participate in OD (Alvaro et al. 2008; Saleem et al. 2009; Bratton et al. 2011). The authors found that people looked online, on television, radio and other media, as well as healthcare establishments, for information. In another study, it appeared initially that the subjects knew a great deal about OD (Krupic et al. 2018). It is good for people to find information by themselves about both OD and the healthcare system. However, the provision of the class meant that information about their faith and OD and the healthcare system was more accessible to them and they were therefore more likely to participate in OD in some way. Our previous study showed that the most important factors affecting OD were the lack of information in society and from health care (Krupic et al. 2017). All the subjects in the study were well educated, from different countries and were well integrated into Swedish life. They all spoke several languages, were employed and in contact with many different people on a daily basis. They all used the Internet regularly. They all stated that more information about organ donation and the healthcare system will lead to more people being willing to be organ donors. They also mentioned that it is important to have a donor card. This has also been shown to be influenced by their religious education (Krupic et al. 2018), which only partially agrees with the present study. In this study, the subjects had not received good education regarding their religion, but, after only a 4-h class about their religion and the healthcare system, they all changed their minds and decided to become organ donors. They all repeated that their attitude had changed completely and that they were thankful to God and the teachers for the good, detailed class. They stated how important this class is and that is should be held more often. Unfortunately, due to the state of the healthcare system in Sweden, where professionals have very little time to explain procedures to patients or for patients to ask questions, it is difficult to implement these changes. The fact that the patient is a foreigner and may need help with the language only makes this situation worse. There is always a need to save money, but in the end it is the patients who suffer.

### Limitations

To the best of the author’s knowledge, this is the first study of its kind. However, the present study has some limitations. The interviews were held in mixed groups, with subjects from six different countries and of both genders, which may have made the participants nervous, making it difficult for them to concentrate during the class, the interview and the discussion. Another limitation may be that the interviewer is from the same ethnic group as the subjects from Bosnia and Herzegovina, which may be regarded as a risk factor for impartiality in the planning, execution and analysis of the research, because of pre-conceptions.

## Conclusion

The findings in this study indicate that a class dealing with religion, the religious aspects of organ donation and the way the Swedish healthcare system is organised increased people’s knowledge and changed their attitudes so they became potential organ donors. For even greater success, those who are already better educated about their faith, OD and the healthcare system should be targeted. We also suggest setting up agencies to carry out education to provide more information about religion and the healthcare system. More intervention studies are needed in every field of medicine to build confidence and give time to educate and discuss issues with potential organ donors in Sweden.
